# Risk prediction models with incomplete data with application to prediction of estrogen receptor-positive breast cancer: prospective data from the Nurses' Health Study

**DOI:** 10.1186/bcr2110

**Published:** 2008-07-03

**Authors:** Bernard Rosner, Graham A Colditz, J Dirk Iglehart, Susan E Hankinson

**Affiliations:** 1 Channing Laboratory, Department of Medicine, Brigham and Women's Hospital and Harvard Medical School, 181 Longwood Ave., Boston, MA 02115, USA; 2 Department of Biostatistics, Harvard School of Public Health, 655 Huntington Ave., Boston, MA 02115, USA; 3 Alvin J Siteman Cancer Center and Department of Surgery, Washington University School of Medicine, Campus Box 8100, 660 S. Euclid Ave., St. Louis, MO 63110, USA; 4 Department of Cancer Biology, Dana Farber-Cancer Institute and Harvard Medical School and Department of Surgery, Brigham and Women's Hospital and Harvard Medical School, 44 Binney Street, Boston, MA 02115, USA; 5 Department of Epidemiology, Harvard School of Public Health, 677 Huntington Ave., Boston, MA 02115, USA

## Abstract

**Introduction:**

A number of breast cancer risk prediction models have been developed to provide insight into a woman's individual breast cancer risk. Although circulating levels of estradiol in postmenopausal women predict subsequent breast cancer risk, whether the addition of estradiol levels adds significantly to a model's predictive power has not previously been evaluated.

**Methods:**

Using linear regression, the authors developed an imputed estradiol score using measured estradiol levels (the outcome) and both case status and risk factor data (for example, body mass index) from a nested case-control study conducted within a large prospective cohort study and used multiple imputation methods to develop an overall risk model including both risk factor data from the main cohort and estradiol levels from the nested case-control study.

**Results:**

The authors evaluated the addition of imputed estradiol level to the previously published Rosner and Colditz log-incidence model for breast cancer risk prediction within the larger Nurses' Health Study cohort. The follow-up was from 1980 to 2000; during this time, 1,559 invasive estrogen receptor-positive breast cancer cases were confirmed. The addition of imputed estradiol levels significantly improved risk prediction; the age-specific concordance statistic increased from 0.635 ± 0.007 to 0.645 ± 0.007 (*P *< 0.001) after the addition of imputed estradiol.

**Conclusion:**

Circulating estradiol levels in postmenopausal women appear to add to other lifestyle factors in predicting a woman's individual risk of breast cancer.

## Introduction

Breast cancer risk prediction models have been developed for use as an entry criterion into breast cancer chemoprevention trials (for example, National Surgical Adjuvant Breast and Bowel Project tamoxifen trial and the Study of Tamoxifen and Raloxifene), in counseling women on the potential use of chemopreventives, and to provide insight into a woman's individual breast cancer risk [1-4]. The initial Gail model incorporated a subset of breast cancer risk factors, namely age, age at menarche, age at first birth, family history of breast cancer or of atypical hyperplasia, and history of breast biopsies [5,6]. Subsequently, several groups have developed more extensive statistical models that incorporate a greater number of breast cancer risk factors [1,4].

In postmenopausal women, circulating levels of estradiol predict subsequent breast cancer risk [7-10], particularly for estrogen receptor (ER)-positive disease [9]. However, plasma estradiol levels have not previously been evaluated within risk prediction models, and whether their addition would add to the model's predictive power is unknown. Plasma estradiol is available only from the Nurses Health Study nested case-control data set. We initially attempted to evaluate the addition of estradiol concentrations to the Rosner and Colditz risk prediction model using data from the Nurses' Health Study nested case-control data set. However, with the relatively modest size of the nested case-control data set and the large number of parameters to be estimated, a number of the risk factor parameters did not adequately reflect those from the parent cohort. Thus, development of an accurate risk prediction tool requires a large sample size as in the main Nurses' Health Study cohort. The purpose of this paper is to describe a methodology for developing a risk prediction rule when one or more predictors are incompletely observed and to apply it to assess the predictive power of plasma estradiol after adjusting for standard breast cancer risk factors as well as the effect of other risk factors adjusted for plasma estradiol.

## Materials and methods

### Cohort

The Nurses' Health Study cohort was established in 1976, when 121,700 female US nurses (30 to 55 years old) responded to a mailed questionnaire that inquired about reproductive history and a range of lifestyle factors in addition to disease diagnoses [11,12]. Follow-up questionnaires have been mailed biennially to update exposure information and any major medical events. Deaths are reported by family members or the postal service or are identified by a search of the National Death Index. We estimate that mortality ascertainment is 98% complete [13,14]. This investigation was approved by the Brigham and Women's Hospital institutional review board.

### Identification of breast cancer cases

On each questionnaire, we inquired whether breast cancer had been diagnosed and, if so, the date of diagnosis. All women who reported breast cancer (or the next of kin for decedents) were contacted for permission to review medical records to confirm the diagnosis. We include only invasive cases of breast cancer confirmed by the pathology report. ER and progesterone receptor status of the tumor was determined from the medical record. In this report, we evaluate ER^+ ^cases only, as we previously observed that plasma estradiol most strongly predicted this tumor subtype [9].

### Population for analysis

The population of women used in this analysis has been described in detail in several previous publications [1,15]. Briefly, we excluded women with unknown, inconsistent, or out-of-range reports for height, weight in 1976 or at age 18, age at menarche or menopause or each pregnancy, parity, and duration or type of postmenopausal hormone (PMH) use (n = 42,886). Additionally, women with a simple hysterectomy (and hence unknown age at menopause) (n = 10,301) were excluded. Participants who were ineligible for the study (for example, prevalent cancer in 1976) or no follow-up after 1978 (n = 2,360) were excluded. In the current analysis, women who were premenopausal throughout follow-up were excluded (n = 6,342), but once they became postmenopausal, they could contribute person-time. Overall, 59,812 participants remained for this analysis. These women contributed 750,086 person-years from 1980 to 2000, during which 1,559 incident invasive ER^+ ^breast cancer cases occurred.

### Blood subcohort and nested case-control study

From 1989 to 1990, 32,826 cohort members provided blood samples. Informed consent was obtained from each participant; details about the blood collection methods have been published previously [16,17]. Briefly, women arranged to have their blood drawn and shipped with an icepack via overnight courier to our laboratory, where it was processed and archived in liquid nitrogen freezers. Estradiol is stable in cooled whole blood for 24 to 48 hours [18]. At blood collection, women completed a short questionnaire that included questions on recent use of PMH (within the last 3 months). Follow-up of the blood study cohort was 99% in 2000.

In the current analyses, we used a previously described nested case-control study of sex steroids and breast cancer risk with cases diagnosed after blood collection through 31 May 1998 [9,16]. In addition, cases diagnosed up through 31 May 2000 and their matched controls (that is, a 2-year extension of the published report [9]) are included. At blood collection, cases and controls were postmenopausal, were not recent users of PMH, and had no prior diagnosed cancer (except nonmelanoma skin cancer). Control subjects were matched by age, month/year and time of day of blood collection, and fasting status and had not been diagnosed with breast cancer before the diagnosis date of their matched case. To mimic the larger population used in the risk prediction modeling, only cases and controls meeting the inclusion criteria described above were included (for example, no prior simple hysterectomy). Women were considered postmenopausal if they reported having a natural menopause (for example, no menstrual cycles during the previous 12 months) or had a bilateral oophorectomy. In all, 164ER^+ ^cases and 346 controls were included.

### Laboratory assays

Estradiol was measured by radioimmunoassay following extraction and celite column chromatography, as previously described [9]. The coefficient of variation was less than or equal to 11%.

### Description of the risk prediction model

We fit the log-incidence model of breast cancer to incident ER^+^cases, as previously described [1,15]. We assume that incidence at time, *t*(*I*_*t*_), is proportional to the number of cell divisions, *C*_*t*_, accumulated throughout life up to age *t*; that is,

(1) *I*_*t *_= *kC*_*t*_

The cumulative number of breast cell divisions is factored as follows:

(2) $${\text{C}}_{\text{t}}={\text{C}}_{\text{o}}\text{x}\prod_{\text{i}=0}^{\text{t}-1}\left({\text{C}}_{\text{i}+1}/{\text{C}}_{\text{i}}\right)={\text{C}}_{\text{o}}\text{x}\prod_{\text{i}=0}^{\text{t}-1}\lambda_{\text{i}}$$

Thus, *λ*_*i *_= *C*_*i*+1_/*C*_*i *_represents the rate of increase of breast cell divisions from age *i *to age *i*+1. Log (*λ*_*i*_) is assumed to be a linear function of risk factors that are relevant at age *i*. The set of risk factors and their magnitude may vary according to the stage of reproductive life. Details of the representation of the *C*_*i *_are given in [1,15]. The overall model is given by:

(3) $$\begin{array}{l} \log I=\alpha +\beta_{0}(t_{m}-t_{0})\\ +\beta_{1}b+\beta_{2}(t_{1}-t_{0})b_{1,t-1} \end{array}$$

+γ1(t−tm)mA+γ2(t−t)mmB+δ1 pmhA+δ2 pmhB+δ3 pmhC+δ4 pmhcur,t+(δ4+δ5) pmhpast,t+β3BMI1+β3*BMI2+β4h1+β4*h2+α1 bbd+α2 bbd to+α3 bbd (tm−to)+α4 bbd (t−tm)mt+φ fhx+β5 alc1+β5∗ alc2+β5∗∗ alc3

*t *= age

*t*_*o *_= age at menarche

*t*_*m *_= age at menopause

*s*_*t *_= parity at age *t*

*t*_*i *_= age at *i*th birth, *i *= 1, ..., *s*_*t*_

*b *= birth index = $\sum_{i=1}^{s}(t_{m}-t_{i})b_{it}$ for parous women, = 0 for nulliparous women

*b*_*it *_= 1 if parity is greater than or equal to *i *at age *t *(otherwise, *b*_*it *_= 0)

*m*_*A *_= 1 if natural menopause (otherwise, *m*_*A *_= 0)

*m*_*B *_= 1 if bilateral oophorectomy (otherwise,*m*_*B *_= 0)

*bbd *= 1 if breast disease is benign (otherwise, *bbd *= 0)

*fhx *= 1 if there is a family history of breast cancer in mother or sister (otherwise, *fhx *= 0)

*pmh*_*A *_= number of years on oral estrogen

*pmh*_*B *_= number of years on oral estrogen and progestin

*pmh*_*C *_= number of years on other types of PMHs

*pmh*_*cur*,*t *_= 1 if current user of postmenopausal hormones at age *t *(otherwise, *pmh*_*cur*,*t *_= 0)

*pmh*_*past*,*t *_= 1 if past user of postmenopausal hormones at age *t *(otherwise, *pmh*_*past*,*t *_= 0)

*BMI*_*j *_= body mass index (BMI) at age *j *(kg/m^2^)

*alc*_*j *_= alcohol consumption (grams) at age *j*

*h *= height (inches)

$$BMI_{1}=\sum_{j=t_{o}}^{t_{m}-1}\left(BMI_{j}-21.8\right)+\sum_{j=t_{m}}^{t-1}(BMI_{j}-24.4)pmh_{cur,j}$$

$$BMI_{2}=\sum_{j=t_{m}}^{t-1}\left(BMI_{j}-24.4)\;(1-pmh_{cur.j}\right)$$

$$h_{1}=(h-64.5)(t_{m}-t_{o})+(h-64.4)\sum_{j=t_{m}}^{t-1}pmh_{cur,j}$$

$$h_{2}=(h-64.4)\sum_{j=t_{m}}^{t-1}\left(1-pmh_{cur,j}\right)$$

$$alc_{1}=\sum_{j=18}^{t_{m}-1}alc_{j}$$

$$alc_{2}=\sum_{j=t_{m}}^{t-1}alc_{j}pmh_{cur,j}$$

$$alc_{3}=\sum_{j=t_{m}}^{t-1}alc_{j}(1-pmh_{cur,j}).$$

The general rationale for a log-incidence model is that the number of precancerous cells increases multiplicatively with time but that historical exposures differentially affect the rate of increase. Specifically, for breast cancer, the number of precancerous cells is assumed to increase annually at the rate of exp(*β*_0_) prior to menopause for nulliparous women, at the rate of exp(*β*_0 _+ *β*_1_s) prior to menopause for parous women with parity = s, and so forth. Finally, the number of precancerous cells increases immediately after the first birth by exp [*β*_2_(t_1 _- t_0_)]. The incidence rate of breast cancer is assumed to be approximately proportional to the number of precancerous cells.

The log-incidence model was fit using iteratively reweighted least squares with PROC NLIN in SAS (SAS version 6.12; SAS Institute Inc., Cary, NC, USA) (1996). The parameters of the model are readily interpretable in a relative risk (RR) context. For example, exp(-*β*_0_) = RR for a 1-year increase in age at menarche among nulliparous women, exp [-(*β*_0 _+ *β*_2_)] = RR for a 1-year increase in age at menarche among parous women, and so forth. In this analysis, women were followed until they had an event (ER^+ ^breast cancer) or were censored if they developed (a) ER^- ^breast cancer, (b) breast cancer in which ER status is unknown, or (c) other types of cancer except nonmelanoma skin cancer or (d) if they died.

### Imputation and inclusion of estradiol in the risk prediction model

Ideally, we would have estradiol levels measured on each main study participant at several points in time. However, since this was not possible, we used an indirect approach to impute estradiol. Let *x *= estradiol and *z* = other covariates in the risk prediction model.

From the main study, we can obtain Pr(D|z) given under the rare disease assumption by:

(4) $$\text{Pr(D|}\underline{\text{z}}\text{)}=\text{exp(}\alpha +\underline{\beta }\underline{\text{z}}\text{)/[1}+\text{exp(}\alpha +\underline{\beta }\underline{\text{z}}\text{)]}≅\exp(\alpha +\underset{˜}{\beta }\underset{˜}{z}).$$

We want to estimate Pr(*D*|*x*,*z*), where under the rare disease assumption

(5) Pr(*D*|*x*,*z*) ≅ exp(α* + β*z + δ*x).

From the blood study, we can estimate δ* based on conditional logistic regression. Indeed, in principle, we could also estimate β* from the blood study, but the estimates will be very imprecise due to the small sample size. Therefore, we used the main study population to estimate the parameters in Equation 5 by estimating *x *for all subjects in the main study based on a linear regression derived from the blood study:

(6) *x *= *α*_*o *_+ *γ*_*o*_*y *+ *γ**z*_*imp *_+ *e*,

where *x *= ℓn (estradiol) as a continuous variable, y = 1 if case and 0 if control, and *Z*_*imp *_= a subset of the other covariates *Z* in the risk prediction model. *Z*_*imp *_was ascertained by first forcing in *y *and then using stepwise-up regression to determine the subset of components of *Z* in the main study which were significantly associated with *x *at the 5% level.

In the blood study, estradiol levels on average were higher for cases than controls. The rationale for including *y *as a covariate in Equation 6 is to account for this relationship in the main study as well. In addition, because there is substantial overlap between the estradiol distribution of cases and controls, we used an imputation strategy to estimate *x *by adding error to the prediction such that for each main study participant we obtain

(7) $${\hat{x}}_{i}=\alpha_{o}+\gamma_{o}y_{i}+\underline{\gamma }{\underline{z}}_{imp,i}+e_{i},$$

where (a) *e*_*i *_= an error term that is normally distributed with mean 0 and variance σ^2^, (b) y_*i *_= 1 if a breast cancer case and = 0 otherwise, (c) σ^2 ^is estimated from Equation 6, and *e *is obtained by the RANNOR function of SAS so as to add error to the estimate of *x *for individual women. We then fit the model in Equation 5 using $\hat{x}$ instead of *x*, thus obtaining the model

(8) $$\mathrm{Pr}(D|x,z)≅\exp(\alpha **+\beta **z+\delta **\hat{x}).$$

Since the parameter estimates in Equation 8 may be influenced by the random error introduced in Equation 7, we repeated this imputation approach four additional times and used multiple imputation [19] to combine estimates from the separate imputations to obtain an overall estimate.

To assess the additional predictive power of serum estradiol, we computed age-specific (5-year age groups) deciles of the risk function without estradiol (model A) as well as including imputed estradiol (model B). From the cross-classification of risk decile model A × risk decile model B, we then compared the observed number of cases in specific risk deciles of model B with the expected number of cases within strata defined by model A risk decile. Specifically, let X_ij _= the number of breast cancer cases, N_ij _= the number of person-years, and p_ij _= X_ij_/N_ij_, which is the estimated incidence rate within the ith age-specific risk decile for model A and the jth age-specific risk decile for model B, and let ln(p_ij_) = α_i _+ β(j - 1). 100% × [exp($\overset{⌢}{\beta }$)-1] is an estimate of the percentage increase in breast cancer incidence for an increase of one model B risk decile, holding the model A risk decile constant [20]. We wish to test the hypothesis H_0_: β = 0 versus H_1_: β ≠ 0. This approach of cross-classifying individuals by two different risk prediction rules is similar to the reclassification table approach used to compare risk prediction rules in the Framingham Heart Study [21]. In addition, to assess the predictive ability of our risk prediction models, we used the area under the receiver operating characteristic curve (that is, the concordance or C statistic). This statistic ranges from 0.5 to 1.0 and represents the probability that, for a randomly selected pair of women, one with ER^+ ^breast cancer and one without breast cancer, the woman with ER^+ ^breast cancer has the higher estimated disease probability. Also, we compared the C statistic for different risk prediction rules [22]. In our primary analysis, we evaluated the addition of imputed estradiol levels to risk prediction models in the entire cohort. As a secondary approach, we calculated Rosner and Colditz model risk scores in the entire cohort and then, in the nested case-control data set, assessed the impact of adding this score to the plasma estradiol and breast cancer model.

## Results

Within the nested case-control data set, we observed a significant association between plasma estradiol and risk of breast cancer (*P*_trend _< 0.001), with an RR for the top (Q4) versus bottom (Q1) quartile category of 3.3 (95% confidence interval [CI] = 1.8 to 6.0) for ER^+ ^breast cancer (Table 1). Each of the variables in the Rosner and Colditz risk prediction model was considered as a potential predictor of plasma estradiol. BMI was most strongly related to estradiol level; in addition, the birth index, case status, and duration of postmenopause each contributed modestly but significantly (Table 2). Other variables, including family history of breast cancer, alcohol intake, and history of BBD, did not contribute significantly to the model and thus were dropped from further consideration. The *r*^2^, for the regression model, was 0.219.

**Table 1:** Relative risk of estrogen receptor-positive breast cancer by quartile of postmenopausal plasma estradiol concentration (164 cases and 346 controls)^a^

	Quartile of plasma estradiol				
	1	2	3	4	*P *for trend

Relative risk	1	1.6	1.4	3.3	
95% confidence interval		0.9–2.9	0.8–2.7	1.8–6.0	<0.001

^a^Unconditional logistic regression controlling for matching factors (age, month and time of day of blood collection, and fasting status) and duration of premenopause, duration of menopause (separately for natural menopause and bilateral oophorectomy), birth index, age at first birth minus age at menarche, benign breast disease, duration of estrogen use, duration of estrogen and progestin use, duration of use of other types of postmenopausal hormones (PMH), current PMH use, past PMH use, average body mass index before and after menopause, and family history of breast cancer.

**Table 2:** Imputation of log estradiol using data from the Nurses' Health Study breast cancer nested case-control study^a^

Variable	Beta	Standard error	*P *value
Intercept	2.241	0.085	
Case status	0.130	0.048	0.008
Body mass index (postmenopausal)^b^	0.0033	0.0003	<0.001
Birth index^c^	-0.0021	0.0006	<0.001
Duration of natural menopause	-0.015	0.004	<0.001
Duration of menopause after bilateral oophorectomy	-0.013	0.004	0.004
*R*^2 ^= 0.219			

^a^Based on 164 cases and 346 controls. ^b^Duration postmenopause × average body mass index postmenopause. ^c^Birth index = $\sum_{i=1}^{s}(t_{m}-t_{i})b_{it}$ if parous and = 0 if nulliparous where *s *= parity; *t*_*i *_= age at *i*th birth, *i *= 1, ..., *s*; *t*_*m *_= age at menopause; and *b*_*it *_= 1 if parity is greater than or equal to *i *at age *t *and = 0 otherwise.

Most variables in the Rosner and Colditz model incorporate a time component (for example, postmenopausal BMI = average BMI postmenopause × duration postmenopause). Because exposure status at the time of blood draw might be most strongly correlated with estradiol, we also evaluated each of the variables at the time of blood draw or, for variables ascertained only on the main study questionnaire (for example, alcohol intake), within 2 years of blood draw. All results were similar.

In Table 3, we present the standard risk prediction model without estradiol and an enhanced model with imputed estradiol based on an average of five imputations. The regression coefficient for log_e _estradiol varied over the five imputations (0.400 to 0.582) with an average of 0.477. For a one-unit increase in log_e _estradiol, the RR was 1.61 (95% CI = 1.35 to 1.93). Its inclusion also caused changes in some of the other model parameters. As expected, the regression coefficient for postmenopausal BMI decreased from 0.0038 (*P *< 0.001) to 0.0023 (*P *= 0.002). For example, if we compare two 70-year-old postmenopausal women with age at menopause of 50 years, no PMH use, and constant BMI of 20 and 30, respectively, from age 50 to 70, then the RRs of breast cancer at age 70 for the woman with BMI = 30 versus the woman with BMI = 20 would be exp(0.0038 × 10 × 20) = 2.1 (95% CI = 1.7 to 2.7) without controlling for estradiol and exp(0.0023 × 10 × 20) = 1.6 (95% CI = 1.2 to 2.1) after controlling for estradiol. Also, the regression coefficient for the birth index decreased from -0.0030 ± 0.0007 (*P *< 0.001) to -0.0020 ± 0.0008 (*P *= 0.02). To interpret this difference, we compare two postmenopausal women with age at menarche of 13 and age at menopause of 50, one of whom had four births at ages 20, 23, 26, and 29 whereas the other was nulliparous. The birth indices for these two women are 102 [(50 - 20) + (50 - 23) + (50 - 26) + (50 - 29)] and 0, respectively. The RRs for the parous versus nulliparous woman are exp [-0.0030(102)] = 0.74 (95% CI = 0.64 to 0.85) without and exp [-0.0020(102)] = 0.82 (95% CI = 0.69 to 0.96) with controlling for log_e _estradiol. Finally, the regression coefficient for both the duration after natural menopause and the duration after bilateral oophorectomy increased after adjusting for log_e _estradiol. To interpret this finding, we compare two postmenopausal women with age at natural menopause of 45 and 55 years, respectively. The RRs of breast cancer for the second compared with the first woman are exp [10(0.102-0.049)] = 1.7 (95% CI = 1.4 to 2.0) without and exp [10(0.101-0.056)] = 1.6 (95% CI = 1.3 to 1.9) with adjusting for log_e _estradiol. It appears that part of the effect of BMI, parity, and late menopause on the incidence of breast cancer is mediated in part by changes in log_e _estradiol caused by obesity (increase), multiparity with first birth at an early age (decrease), and delayed menopause (increase), respectively.

**Table 3:** Risk prediction model for breast cancer with and without estradiol (log_e_)

No estradiol model						
Parameter	Beta	SE	*P *value	Increment	RR	95% CI

Intercept	-10.8					
Duration of premenopause, years	0.102	0.009	<0.001	1	1.11	1.09, 1.13
(Age at first birth minus age at menarche) × parous^a^	0.0035	0.0051	0.49	10	1.04	0.94, 1.14
Birth index^a^	-0.0030	0.0007	<0.001	102	0.74	0.64, 0.85
Duration of postmenopause (natural menopause)	0.049	0.006	<0.001	1	1.05	1.04, 1.06
Duration of postmenopause (bilateral oophorectomy)	0.039	0.008	<0.001	1	1.04	1.02, 1.06
Benign breast disease	0.288	0.656	0.66	1	1.33	0.37, 4.82
Benign breast disease × age at menarche	0.091	0.027	0.001	1	1.10	1.04, 1.15
Benign breast disease × duration premenopause	-0.018	0.012	0.14	1	0.98	0.96, 1.01
Benign breast disease × duration postmenopause	-0.029	0.008	<0.001	1	0.97	0.96, 0.99
Duration of estrogen use	0.032	0.008	<0.001	10	1.38	1.18, 1.61
Duration of estrogen plus progestin use	0.068	0.013	<0.001	10	1.97	1.53, 2.55
Duration of other PMH use	0.032	0.011	0.006	10	1.38	1.11, 1.71
Current PMH use	0.109	0.079	0.17	1	1.12	0.96, 1.30
Past PMH use	-0.022	0.073	0.76	1	0.98	0.85, 1.13
Average BMI premenopause or postmenopause while on PMH × (duration premenopause + duration postmenopause while on PMH)	-0.0011	0.0003	<0.001	370^b^	0.67	0.54, 0.83
Average BMI postmenopause while not on PMH × (duration postmenopause while not on PMH)	0.0038	0.0006	<0.001	200^c^	2.14	1.69, 2.71
Height × (duration premenopause + duration postmenopause while on PMH)	0.00072	0.00037	0.052	222^d^	1.17	1.00, 1.38
Height × (duration postmenopause while not on PMH)	-1 × 10^-5^	0.0015	0.99	120^e^	1.00	0.70, 1.42
Cumulative alcohol consumption before menopause, grams	0.00034	0.00009	<0.001	384^f^	1.14	1.06, 1.22
Cumulative alcohol consumption after menopause while on PMH	-0.00036	0.00041	0.39	120^g^	0.96	0.87, 1.05
Cumulative alcohol consumption postmenopause while not on PMH	0.00008	0.00031	0.80	240^h^	1.02	0.88, 1.18
Family history of breast cancer	0.447	0.065	<0.001	1	1.56	1.38, 1.78
						
Model including log_e _estradiol^i^						

Parameter	Beta	SE	*P *value	Increment	RR	95% CI

Intercept	-11.9					
Duration of premenopause, years	0.101	0.01	<0.001	1	1.11	1.08, 1.13
(Age at first birth minus age at menarche) × parous^a^	0.0035	0.0055	<0.001	10	1.04	0.93, 1.15
Birth index	-0.0020	0.0008	0.02	102	0.82	0.69, 0.96
Duration of postmenopause (natural menopause)	0.056	0.007	<0.001	1	1.06	1.04, 1.07
Duration of postmenopause (bilateral oophorectomy)	0.045	0.009	<0.001	1	1.05	1.03, 1.06
Benign breast disease	0.287	0.719	0.69	1	1.33	0.33, 5.45
Benign breast disease × age at menarche	0.091	0.029	0.002	1	1.10	1.03, 1.16
Benign breast disease × duration premenopause	-0.018	0.014	0.18	1	0.98	0.96, 1.01
Benign breast disease × duration postmenopause	-0.029	0.008	0.001	1	0.97	0.96, 0.99
Duration of estrogen use	0.032	0.009	<0.001	10	1.38	1.15, 1.64
Duration of estrogen plus progestin use	0.068	0.014	<0.001	10	1.97	1.50, 2.60
Duration of other PMH use	0.032	0.013	0.012	10	1.38	1.07, 1.78
Current PMH use	0.110	0.087	0.21	1	1.12	0.94, 1.32
Past PMH use	-0.022	0.080	0.78	1	0.98	0.84, 1.14
Average BMI premenopause or postmenopause while on PMH × (duration premenopause + duration postmenopause while on PMH)	-0.0011	0.0003	<0.001	370^b^	0.67	0.54, 0.83
Average BMI postmenopause while not on PMH × (duration postmenopause while not on PMH)	0.0023	0.0007	0.002	200^c^	1.58	1.20, 2.08
Height × (duration premenopause + duration postmenopause while on PMH)	0.00072	0.00041	0.072	222^d^	1.17	0.98, 1.40
Height × (duration postmenopause while not on PMH)	0	0.0016	1	120^e^	1.00	0.69, 1.46
Cumulative alcohol consumption before menopause, grams	0.00034	0.0001	0.001	384^f^	1.14	1.06, 1.23
Cumulative alcohol consumption after menopause while on PMH	-0.00036	0.00045	0.43	120^g^	0.96	0.86, 1.06
Cumulative alcohol consumption postmenopause while not on PMH	0.00008	0.00034	0.81	240^h^	1.02	0.87, 1.20
Family history of breast cancer	0.446	0.072	<0.001	1	1.56	1.36, 1.80
Log_e _estradiol	0.477	0.092	<0.001	1	1.61	1.35, 1.93

^a^0 for nulliparous women.

^b^Comparing BMI = 20 versus BMI = 30 for 37 premenopausal years (age at menarche = 13, age at menopause = 50).

^c^Comparing BMI = 20 versus BMI = 30 for 20 postmenopausal years (age at menopause = 50, age = 70).

^d^Comparing height = 61 inches versus height = 67 inches for 37 premenopausal years (age at menarche = 13, age at menopause = 50).

^e^Comparing height = 61 inches versus height = 67 inches for 20 postmenopausal years (age at menopause = 50, age = 70).

^f^Comparing 1 drink per day (12 g) versus no intake for 32 premenopausal years (from age 18 to age 50).

^g^Comparing 1 drink per day (12 g) versus no intake for 10 years of PMH use.

^h^Comparing 1 drink per day (12 g) versus no intake for 20 postmenopausal years (age at menopause = 50, age = 70).

^i^Based on an average of five imputations.

BMI, body mass index; CI, confidence interval; PMH, postmenopausal hormone; RR, relative risk; SE, standard error.

We now present the cross-classification of model A × model B risk decile in Table 4. It is clear from Table 4 that, within most model A risk deciles, there are important differences in estimated incidence according to model B risk decile (often twofold). Overall, for a given model A risk decile, the observed number of cases was higher than expected when the model B decile was high and lower than expected when the model B decile was low. The overall slope was β = 0.511 ± 0.034 (*P *< 0.001), indicating that there is a significant estimated 67% increase in breast cancer incidence for an increase of one model B age-specific risk decile, holding the age-specific model A risk decile constant. This indicated that there is substantial increased predictive power upon adding log_e _estradiol to the risk prediction model.

**Table 4:** Cross-classification of model A risk decile^a ^× model B risk decile^b^

Model B risk decile													
	1	2	3	4	5	6	7	8	9	10	Slope	SE	*P *value

Model A risk decile	Cases/py	Cases/py	Cases/py	Cases/py	Cases/py	Cases/py	Cases/py	Cases/py	Cases/py	Cases/py			

	(Rate)	(Rate)	(Rate)	(Rate)	(Rate)	(Rate)	(Rate)	(Rate)	(Rate)	(Rate)			

1	41/61,883	13/12,253	0/756	0/20	0/2	0/0	0/0	0/0	0/0	0/0	0.471	0.318	0.14
	(66)	(106)	(0)	(0)	(0)	(-)	(-)	(-)	(-)	(-)			
2	9/12,317	34/42,222	36/17,656	7/2,535	1/187	0/6	0/0	0/0	0/0	0/0	0.593	0.129	<0.001
	(73)	(81)	(204)	(276)	(535)	(0)	(-)	(-)	(-)	(-)			
3	0/694	9/17,749	46/34,208	31/18,339	8/3,678	1/241	0/8	0/0	0/0	0/0	0.396	0.126	0.002
	(0)	(51)	(134)	(169)	(218)	(415)	(0)	(-)	(-)	(-)			
4	0/18	1/2,567	18/18,588	42/31,754	43/18,223	13/3,547	1/217	0/6	0/0	0/0	0.48	0.102	<0.001
	(0)	(39)	(97)	(132)	(236)	(367)	(461)	(0)	(-)	(-)			
5	0/0	0/132	5/3,477	13/18,417	47/31,547	50/18,268	18/3,010	2/158	0/0	0/0	0.565	0.085	<0.001
	(-)	(0)	(144)	(71)	(149)	(274)	(598)	(1,000)	(-)	(-)			
6	0/0	0/2	1/231	3/3,635	21/18,333	50/32,782	54/17,504	13/2,363	0/70	0/0	0.469	0.088	<0.001
	(-)	(0)	(433)	(83)	(68)	(153)	(309)	(550)	(0)	(-)			
7	0/0	0/0	0/2	0/210	2/2,922	22/17,752	76/35,260	46/17,428	9/1,351	0/0	0.44	0.099	<0.001
	(-)	(-)	(0)	(0)	(68)	(124)	(216)	(264)	(666)	(-)			
8	0/0	0/0	0/0	0/10	0/116	4/2,272	31/17,599	87/35,817	79/16,063	1/339	0.5	0.091	<0.001
	(-)	(-)	(-)	(0)	(0)	(176)	(176)	(226)	(492)	(295)			
9	0/0	0/0	0/0	0/0	0/0	0/52	4/1,326	25/16,137	140/46,314	65/11,093	0.553	0.099	<0.001
	(-)	(-)	(-)	(-)	(-)	(0)	(302)	(155)	(302)	(586)			
10	0/0	0/0	0/0	0/0	0/0	0/0	0/0	1/305	21/11,127	315/63,483	0.841	0.207	<0.001
	(-)	(-)	(-)	(-)	(-)	(-)	(-)	(328)	(189)	(496)			
Overall											0.511	0.034	<0.001

^a^Model A, standard risk prediction model without estradiol. ^b^Model B, standard risk prediction model plus imputed estradiol. py, person-years; SE, standard error.

In addition, we compared the age-specific C statistics between model A versus model B. We found C statistics of 0.635 ± 0.007 for model A and 0.645 ± 0.007 for model B (C statistic model A versus C statistic model B; *P *< 0.001). Using our secondary approach (applying the population-based risk scores to the subset of women in the nested case-control study), the RRs of breast cancer by plasma hormone level were similar, though slightly attenuated, compared with those in Table 1. For example, the RRs of ER^+ ^breast cancer with increasing quartile of estradiol were 1.0, 1.5, 1.4, and 2.5 (95% CI = 1.5 to 4.2). Finally, in Table 5, we present the 5-year incidence of breast cancer by age and model B risk decile after adjusting for competing mortality risks [23]. The RR of breast cancer comparing women at the highest versus the lowest age-specific decile ranges from 5.0 to 8.5. For example, for 60- to 64-year-old women, the absolute 5-year risk of breast cancer is 436/10^5^(0.4%) for women in the first decile and 2,982/10^5 ^(3.0%) for women in the 10th decile (RR = 6.8), indicating substantial differences in absolute risk according to the model B risk equation.

**Table 5:** Five-year risk of breast cancer by age and model B risk decile

Model B risk decile											
Age group, years		1	2	3	4	5	6	7	8	9	10

30–34	5-year incidence^a^	65	73	82	97	105	127	147	197	249	321
	RR	1	1.1	1.3	1.5	1.6	2.0	2.3	3.0	3.8	5.0
35–39	5-year incidence^a^	82	107	126	142	160	182	214	259	328	504
	RR	1	1.3	1.5	1.7	2.0	2.2	2.6	3.1	4.0	6.1
40–44	5-year incidence^a^	117	159	191	221	253	294	350	425	527	767
	RR	1	1.4	1.6	1.9	2.1	2.5	3.0	3.6	4.5	6.6
45–49	5-year incidence^a^	177	249	299	347	401	467	550	659	823	1,231
	RR	1	1.4	1.7	2.0	2.7	2.6	3.1	3.7	4.6	7.0
50–54	5-year incidence^a^	266	373	447	522	605	702	819	973	1,204	1,824
	RR	1	1.4	1.7	2.0	2.3	2.6	3.1	3.7	4.5	6.9
55–59	5-year incidence^a^	351	495	595	692	795	913	1,057	1,247	1,542	2,368
	RR	1	1.4	1.7	2.0	2.3	2.6	3.0	3.6	4.4	6.7
60–64	5-year incidence^a^	436	630	759	881	1,009	1,151	1,323	1,548	1,909	2,982
	RR	1	1.4	1.7	2.0	2.3	2.6	3.0	3.6	4.4	6.8
65–69	5-year incidence^a^	533	788	951	1,105	1,265	1,445	1,660	1,941	2,383	3,714
	RR	1	1.5	1.8	2.0	2.4	2.7	3.1	3.6	4.5	7.0
70–74	5-year incidence^a^	635	948	1,160	1,358	1,563	1,790	2,058	2,419	2,991	4,616
	RR	1	1.5	1.8	2.1	2.5	2.8	3.2	3.8	4.7	7.3
75–79	5-year incidence^a^	681	1,077	1,326	1,578	1,827	2,119	2,462	2,954	3,671	5,785
	RR	1	1.6	1.9	2.3	2.7	3.1	3.6	4.3	5.4	8.5

^a^Five-year incidence per 10^5 ^person-years adjusting for competing mortality risks. RR, relative risk.

## Discussion

In the Nurses' Health Study, we found that estradiol levels, as imputed from a nested case-control study within the same cohort, added significantly to the Rosner and Colditz risk prediction model, which already includes most confirmed breast cancer risk factors. There was an increase of 67% in incidence per increase of one model B risk decile, holding model A risk decile constant. The increase in the C statistic was also statistically significant.

Strengths of this study include the large size of the cohort and the large number of available questionnaire-based breast cancer risk factors. Additionally, prospectively assessed estradiol levels were available in a subset of the same women. Through the use of both risk factors and case status in the linear regression, our imputed values as applied to the larger cohort accounted for both the association between hormone level and breast cancer and the correlation between hormone levels and other risk factors already in the risk prediction model.

One limitation of the study was that we did not have measured estradiol levels on all cohort members; however, this is a limitation of all large prospective studies because of the high cost of the assays. In addition, due to our desire to have consistent eligibility criteria throughout, only 164 cases and 346 controls in the nested case-control study met all criteria for the model (for example, known age at menopause). Thus, it was not possible within this small data set to provide a sufficiently precise evaluation of the Rosner and Colditz model (which contains 22 beta coefficients); in our initial attempt to evaluate the model, all beta coefficients had wide CIs. In our secondary analyses, in which we used the risk score within the nested case-control data set, plasma estradiol again contributed significantly to the model. However, the RR for estradiol in the secondary analysis for Q4 versus Q1 was 2.5 (95% CI = 1.5 to 4.2) when controlling for other risk factors using the Rosner-Colditz risk scores versus 3.3 (95% CI = 1.8 to 6.0) when individual risk factors were used within the case-control study. One would expect that the effects of other risk factors are more accurately measured by a single risk score derived from a large cohort study than individual risk factors derived from a relatively small nested case-control data set. More generally, this may indicate better control for confounding in small case-control studies based on risk scores derived from large cohorts versus internal control for confounding based on individual risk factors whose regression coefficients are poorly estimated in small case-control studies. With further follow-up and within a collaboration across several cohorts at other institutions, we plan to re-evaluate the case-control approach.

In imputing estradiol levels, only BMI, the birth index, and duration of postmenopause (in addition to case status) were significant predictors of log_e _(estradiol). The correlation between BMI and estradiol was expected given that aromatization of androgens to estrogens in postmenopausal women occurs in adipose tissue [24]. The association with the birth index, a summary variable representing the number and spacing of pregnancies, has been evaluated less frequently and data are not as consistent [25-28]. The association with duration of postmenopause may be due to declines in estrogen after menopause. Alcohol intake, which previously has been found to correlate with estrogen levels in several studies [29], did not contribute significantly here, which is consistent with our previous report from a subset of the current population showing no correlation with estradiol [17]. To our knowledge, no other lifestyle factors have consistently been shown to predict postmenopausal estradiol levels. The correlation between measured and imputed estradiol was 0.47.

With the inclusion of imputed estradiol, the C statistic increased from 0.635 to 0.645, which is a modest improvement but suggests reasonable discriminatory ability overall. However, the reclassification table approach (Table 4) indicated that a substantial difference in incidence is explained by including imputed estradiol, suggesting that the C statistic may be relatively insensitive to additions of single predictors to risk prediction models [21,30]. However, the relationship of one or a combination of risk factors with disease must be very strong – RRs on the order of 100 to 200 between exposed and unexposed – to serve as a screening tool at the individual level [31-33]. Continued expansion of current models with other risk factors (for example, genetic factors, mammographic density, or cytology from nipple aspirate fluid [34]) may further improve the C statistic. In addition, if chemopreventive agents were developed with few risks (and an acceptable cost-benefit ratio), the need to minimize the false-positive rate would decrease, similar to the use of cholesterol-lowering agents for the prevention of heart disease.

## Conclusion

In summary, our data indicate that circulating estradiol levels in postmenopausal women may contribute significantly to current risk prediction models. Further assessment of estradiol in other studies and of other biomarkers that predict risk is needed to continue to improve our ability to predict breast cancer risk and inform prevention strategies. Similar approaches can be used to incorporate other breast cancer biomarkers in overall risk prediction models.

## Abbreviations

BBD = benign breast disease; BMI = body mass index; C statistic = concordance statistic; CI = confidence interval; ER = estrogen receptor; PMH = postmenopausal hormone; Q1, Q2, Q3, and Q4 = first, second, third, and fourth quartile; RR = relative risk.

## Competing interests

The authors declare that they have no competing interests.

## Authors' contributions

BR, GAC, and SEH contributed to the design of the study and to the analysis and interpretation of the data. JDI contributed to the revision of the manuscript and added important clinical insight. All authors read and approved the final manuscript.
